# CT scan: a benefit that may be evil

**DOI:** 10.5935/1808-8694.20120001

**Published:** 2015-11-20

**Authors:** Fernando de A. Quintanilha Ribeiro

**Affiliations:** a Professor of Faculdade de Medicina da Santa Casa de São Paulo; I would like to thank Dr. Fernão B. Alves da Costa for his help

Some weeks ago, I saw a 14-year old girl in my office, who had been submitted to surgery because of a congenital cholesteatoma. Looking at her chart, I saw that the surgery had been a success and she was bringing me a control computerized tomography (CT scan) six months after the procedure. I noticed she had been submitted to an initial CT before the diagnosis, where a small image could be seen in the tympanic cavity. Another CT showed an enlargement of this image, which led her to surgery. She had a third CT after surgery, to serve as a baseline control for a possible recurrence. And now she is bringing me a fourth six-month control CT. Four CT scans already, and I was about to ask her for another CTs scan for a 1-year control!!!!

Something made me stop and think about it. It might have been my age. Remembering how worry we were, we and the patients, about the radiation of a simple x-ray of the facial sinus or the mastoid in three views… -Careful! It might cause cancer in the future! Such concern and worry faded in time after the CT scan became available. Yes, we could then truly see it, in details. How wonderful! The exam was expensive, but we started having CT scans increasingly more often, in reference centers, teaching hospitals and in the fancier places, and its cost started to drop and drop. Today, it is available everywhere, hospital or lab, all over Brazil. Why think about it? If I don't know what it is, I order a CT scan. Did it work? I order another CT. The health plan does not believe me? I order a CT scan. And nobody talks about radiation. Don't those images in the film, and many are they, irradiate? Sure they do, but how much? I decided to check it out.

Google has everything! As does Medscape or Pubmed. Someone in this world must have the same concern. Some people do, but I found just a handful of studies. One from Dr. David A. Johnson^1^, gastroenterologist, Department Head at the Eastern Virginia School of Medicine, which drew my attention. The author states that the first CT scan came out in 1972, and was put to operation a few years later. The first CT scanner at *Santa Casa de São Paulo* was acquired in 1982. Today, we have two scanners, and we do about 1000 exams per month. I took one scan, and I counted 24 slices. This patient had 4 films, in other words, 96 slices. The most relevant was that the exam was but a sample, because in the so-called digitalized system, the images are millimetric and countless. Resolution improved substantially since 1982, but thanks to more irradiation! In 2002, there were 60 million CTs done in the United States, accounting for 70% of all medical-related exposure to x-rays. We don't have these figures for Brazil.

Irradiation is measured in Grays (mGy), and generally used as sieverts (Sv). One millisievert (mSv) is equal to one milligray (mGy).

Notice that: one front and side chest x-ray emits 0.16 mSv. A screening mammography emits 3.00 mSv. And abdomen CT emits 10.00 in adults and 20.00 in neonates. We still lack accurate data to assess, in the long rung, the incidence of cancer in patients irradiated in CT scans, given that it started to be used about 30 years ago. Nonetheless, we can make some inferences. The irradiation on the Japanese population in the atomic bomb event (67 years ago) was of 5 to 150 mSv, the same experienced by workers in the atomic industry for many years (the same in some CT scans). Many studies have been carried out in these groups, and the percentage of malignant tumors along the years was huge, and known to all. The effects of radiation is cumulative and insidious, and anyone might need to be submitted to CT scans in the future, for serious or even vital reasons. I found other papers, some with mathematical studies to correlate exposure to radiation and the incidence of malignant neoplasia, confirming such direct association^2^, ^3^, ^4^. A study carried out by Dr. David Johnson^1^ among American physicians stands out by the lack of knowledge on the risks to which they are submitting their patients when ordering a CT scan. Even radiologists do not have an accurate idea of the radiation emitted by their exams. I found the same here.

The number of images in a mastoid or paranasal sinuses CT scan is huge and unnecessary. How often have we skipped a number of frames just to concentrate on the mastoid atticus, or on a specific paranasal sinus in a control image exam? Is it possible to do localized scans? Yes, it is! But, we don't do it because there is a protocol to do numerous sequential slices, regardless of clinical need. This protocol could be changed for specific cases, scanning only what is of interest to the physician, exposing the patient to much less radiation.

I don't mean to undermine the value of a CT scan, but shouldn't we be more careful in ordering these scans, making better use of the clinical information and other image exams such as simple x-rays or MRI? Let's consider this. Let's check it, study the issue and be concerned with it. It is very likely that we will not witness the consequences of our current approaches, but this does not release us from our blame, for they may just as well happen.

Let's go back to that girl with the congenital cholesteatoma. To the best of my intentions and, apparently, for her benefit, I wanted to have a proper control of her disease, but without noticing it, I could have been throwing a Hiroshima bomb of her.
